# Bridging the Divide: Integrating Cottonseed Oil Content with Agronomic Trait Improvement in Upland Cotton (*Gossypium hirsutum*)—A Review

**DOI:** 10.3390/plants15050750

**Published:** 2026-02-28

**Authors:** Isah Mansur Aminu, Zeeshan Ahmad, Khadija Kamaluddeen Faruk, Muhammad Iyad Abdullahi, Jingwen Pan, Yan Li, Wei Chen, Jinbo Yao, Shengtao Fang, Shouhong Zhu, Yongshan Zhang

**Affiliations:** 1 State Key Laboratory of Cotton Bio-Breeding and Integrated Utilization, Institute of Cotton Research, Chinese Academy of Agricultural Sciences, Anyang 455000, China; 2 Western Agricultural Research Center, Chinese Academy of Agricultural Sciences, Changji 831100, China; 3 Department of Biological Science, Northwest University, Muhammadu Buhari Way, Kano P.M.B. 3220, Kano State, Nigeria

**Keywords:** cottonseed oil content, agronomic traits, pleiotropy, genomic selection, breeding strategies

## Abstract

Cotton (*Gossypium hirsutum*) is globally cultivated for its high-quality fiber; yet, its seed, rich in oil and protein, offers untapped potential for various applications, including food, feed, and industry. With cottonseed oil gaining renewed attention as a valuable co-product, efforts to enhance oil content must contend with long-standing breeding priorities focused on lint yield and fiber quality. A central challenge lies in the complex and often antagonistic genetic relationships between oil accumulation and key agronomic traits. Notably, negative correlations between seed oil content and fiber yield, as well as the pleiotropic nature of several regulatory genes and Quantitative Trait Loci (QTLs), present significant barriers to dual-trait improvement. This review synthesizes current knowledge on the genetic and molecular interplay between cottonseed oil content and other agronomic traits. We examine the architecture of oil-related QTLs and pleiotropic loci, co-expression patterns of shared transcriptional regulators, and metabolic trade-offs influencing carbon allocation between seed and fiber. Recent advances in genomics, transcriptomics, and systems biology are explored as tools to disentangle these trait interactions. We highlight strategies such as multi-trait genomic selection, CRISPR-based uncoupling of antagonistic loci, and the use of wild and exotic germplasm to overcome linkage drag. By providing an integrative overview of the constraints and opportunities at the intersection of oil and agronomic trait improvement, this review lays the groundwork for the development of dual-purpose cotton ideotypes. We propose a conceptual framework for breeding programs to simultaneously enhance fiber yield and oil productivity in a sustainable and climate-resilient manner.

## 1. Cotton as a Dual-Purpose Crop

Cotton (*Gossypium hirsutum*) has evolved as a dual-purpose crop because its seeds are not only economically valuable as a high-quality fiber crop but also nutritionally valuable as a seed source of oils. Cottonseed is a byproduct of lint production, accounting for approximately 60% of seed cotton, and is an excellent source of oil, protein, and energy [1]. Cottonseed oil is one of the five most widely used vegetable oils in the world, with demand in both food and industrial markets [2]. Due to its high protein content (~23%) and 16–24% oil content, cottonseed is also used as animal feed [3]. This dual-purpose nature strengthens the cotton value chain, integrating both textile and oilseed markets. Nevertheless, breeding programs have traditionally focused on fiber quality, and this aspect has commonly led to the exclusion and eventual decline in the seed quality [4]. With the world agriculture facing the challenge of turning to resource-efficient and sustainable production, crops such as cotton present prospects of boosting per-hectare yields and multiplying farmer earnings, as well as satisfying the growing demand of plant-based oils and proteins [5]. This trend has been observed in the latest interest in cottonseed oil due to sustainability objectives, searches for alternative edible oils, and a need to have resilient crop systems in the face of climatic variability [6]. As the world’s population grows and viable land becomes scarce, one way to intensify agricultural production is to maximize the non-fiber value of fiber crops, such as cotton. Cottonseed oil is valued for its high levels of oxidation stability, balanced proportions of fatty acids (including palmitic, oleic, and linoleic acids), and suitability for use as food, industrial, and biofuel oil [7]. Moreover, rising consumer demand for non-genetically modified organism and vegetable oils has established cottonseed oil as a viable alternative to soybean oil and palm oil, especially in regions with significant cotton cultivation [8]. The integration of oil production into the process of growing cotton not only enhances food security but also enhances the value of the crop, which can also raise the profit levels of both small landholders and commercial farmers. This renewed importance has sparked the need to know what genetic phenomena predispose the accumulation of oil within the plant and how this duality within the home can be genetically optimized in modern breeding programs.

Despite its economic and agronomic potential, efforts to maximize the composition and content of cottonseed oil have faced significant challenges since the complex genetic makeup of the cotton genome presents a difficult challenge to overcome [6,9]. An important concern is that an inverse relationship has often been observed between seed oil content and the agronomic traits of cultivars, including lint yield and fiber quality. The primary reason for this trade-off is competition over assimilates available during boll development, whereby carbon and energy resources are primarily focused on fiber development rather than oil synthesis in the seed [10,11]. Additionally, several quantitative trait loci (QTL) and transcriptional factors associated with oil biosynthesis, such as *GhWRI1* and *GhLEC1*, have demonstrated pleiotropy, inadvertently influencing fiber traits or seed formation [12]. The interweaving of genes controlling oil biosynthesis with other agronomic traits makes it difficult to isolate their individual effects. Usual breeding approaches, which often use phenotypic selection based on lint traits, have inadvertently limited progress made on the genetic level in the traits of seed oils. Therefore, cotton breeding programs have shown little development in producing genotypes with high oil content and combining them with good agronomic characteristics, which has been the practical necessity of detailed breeding designs that will overcome these natural genetic trade-offs. Multi-trait genomic selection, CRISPR-based uncoupling of antagonistic loci, and the use of wild and exotic germplasm are some of the available solutions to the problem of linkage drag. This paper presents an integrative evaluation of the boundaries and opportunities in oil–agronomic trait modification and outlines a conceptual breeding framework for developing dual-purpose cotton ideotypes that enhance both fiber yield and oil content while maintaining sustainability and climate resilience. Evidence-based studies, including CRISPR-mediated *GhFAD2* knockout lines that increase oleic acid content without major agronomic penalties, demonstrate the feasibility of targeted metabolic rewiring in cotton and support the practicality of our proposed framework.

## 2. Overview of Cottonseed Oil Biosynthesis

**Figure 1:**
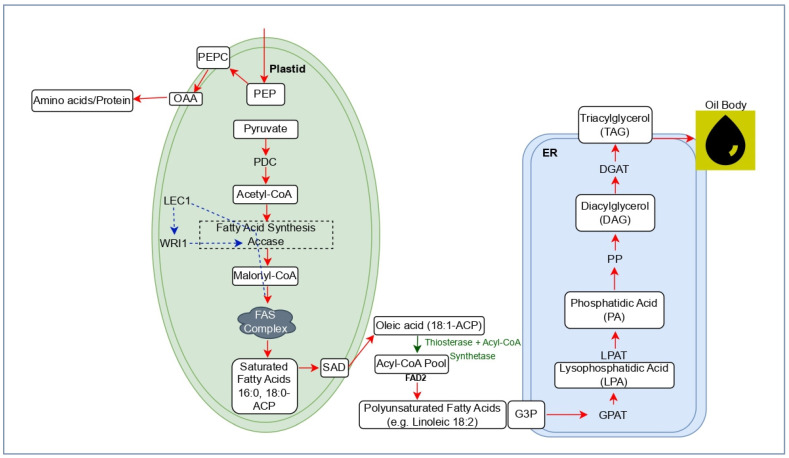
Schematic representation of the cottonseed oil biosynthesis pathway. Solid arrows indicate metabolic flux, dashed arrows indicate regulatory interactions, and background colors denote subcellular compartments (plastid and ER).

Biosynthesis of cottonseed oil begins in the plastids of developing seed cells and ends in the endoplasmic reticulum (ER) through a coordinated process that involves the synthesis of fatty acids, their desaturation, and the assembly of triacylglycerol (TAG) [13] (Figure 1). In cottonseed, the Kennedy pathway is the main pathway for TAG chain synthesis, involving the use of glycerol-3-phosphate to assemble fatty acids into TAGs, which are the primary form of seed oil storage [14,15]. The process starts with the production of acetyl-CoA in the plastids, which is converted to malonyl-CoA by acetyl-CoA carboxylase (ACCase). The fatty acid synthesis (FAS) complex elongates fatty acid chains using malonyl-ACP through sequential condensation reactions, producing mainly palmitic and stearic acids. These saturated fatty acids are exported from the plastids into the cytoplasm and endoplasmic reticulum, where they are combined with glycerol backbones through enzymes of the Kennedy pathway [16]. The final step involves the enzyme diacylglycerol acyltransferase (DGAT), which converts diacylglycerol (DAG) into TAG that forms seed oil bodies [8] (Figure 1). The conversion of stearic acid to oleic and other fatty acids is catalyzed by stearoyl-ACP desaturase (SAD) and fatty acid desaturase 2 (FAD2), resulting in cottonseed oil composition of approximately 25% palmitic acid, 18% oleic acid, and 55% linoleic acid. Modifying desaturase activity, particularly FAD2, enhances oxidative stability and nutritional value, thereby improving oil quality [17]. Although the canonical model of TAG biosynthesis (plastidial acetyl-CoA -malonyl-CoA–FAS–export–ER Kennedy pathway–DGAT) is still a valid biochemical model, both recent multi-omics and functional studies have confirmed and refined this perspective. Functional genome editing of *GhFAD2* (high-oleic edits) and seed-specific expression of WRI1 validate the expected roles of desaturases and LAFL/WRI1 regulators in determining oil composition and accumulation [18,19], thereby confirming foundational targets. However, transcriptomic, metabolomic, and isotope-tracing studies now highlight that flux regulation is often exercised at the level of hexose conversion and plastidial delivery of pyruvate/acetyl-CoA, rather than at the level of individual enzymes, and the practical emphasis of the field has moved toward supply-side nodes [20,21].

Numerous essential enzymes and transcriptional regulators coordinate the movement of carbon in the cottonseed oil biosynthetic pathway. Acetyl-CoA carboxylase (ACCase) initiates plastidial fatty acid production by converting acetyl-CoA into malonyl-CoA, acting as a rate-limiting factor that affects overall oil output [22]. In the ER, FAD2 converts oleic acid to linoleic acid and thereby influences oil stability and nutritional quality; downregulation of FAD2 significantly increases oleic acid content [23]. DGAT catalyzes the terminal step in TAG formation, and its elevated expression correlates with increased oil accumulation in cottonseed [8] (Figure 1). At the transcript level, Leafy Cotyledon1 (LEC1) is a critical developmental regulator that initiates seed development processes, including the activation of wrinkled1 (WRI1), a major transcription factor that enhances glycolytic enzyme activity and regulates fatty acid biosynthesis genes [24]. Collectively, these enzymatic and regulatory components form an integrated network that controls the quantity and composition of cottonseed oil and provides multiple genetic targets to improve yield and quality.

Although this pathway is structurally conserved across cotton, emerging evidence shows that the relative contributions of plastidial fatty-acid synthesis versus ER-based desaturation and TAG assembly can vary among genotypes and environments [25,26]. Genotype-specific changes in ACCase activity, WRI1 expression, or precursor supply affect plastidial flux, whereas quantitative differences in the expression of FAD2, SAD and DGAT influence ER-mediated desaturation and TAG assembly [27]. Additional environmental effects, including heat stress or drought, alter this balance by regulating desaturase expression and plastidial carbon distribution and commonly altering ratios of oleic and linoleic acids [28]. Thus, final oil composition does not only capture the architecture of pathways but also genotype-by-environment variation in plastid and ER metabolic flux.

Cottonseed oil is biosynthetically regulated during development and follows the embryo’s maturation. TAG accumulation commences at approximately 15 Days Post Anthesis (DPA) and peaks between 25 and 35 DPA, coinciding with the transition from globular to cotyledonary embryo stages [29]. This step is characterized by the shifting of metabolism to the compound accumulation at the expense of cell growth as carbon components of photosynthesis are channeled to the biosynthetic mechanism of fatty acids and oils. The time course studies of transcriptome in upland cotton suggests that *WRI1*, *LEC1*, *FUS3*, and *ABI3* reveal the expression profiles that peak at the time of late and mid seed development and are consistent with their role in regulating storage-related gene activation [30,31]. In addition, the use of sugar signaling and hormonal cues (i.e., abscisic acid (ABA) signals) in controlling the regulation of oil biosynthesis genes during this growth stage is vital [32]. It has been suggested that the temporal control mechanisms of oil synthesis, vital genes, involve epigenetic regulation such as histone acetylation and DNA methylation [33]. Recent methylome and chromatin-accessibility analyses in developing cottonseed show dynamic DNA methylation (CG/CHG/CHH) patterns and shifting histone acetylation marks (e.g., H3K9ac) across 10–35 DPA, and these have been correlated with lipid-biosynthesis gene expression [34]. Moreover, experimental manipulation using 5-azacytidine (a DNA methylation inhibitor) in cultured cotton ovules altered expression of key oil-biosynthesis genes (e.g., WRI1, AC-Case), providing functional evidence that methylation status modulates seed oil transcriptional networks [35].

Additional genetic expectations are further elaborated in pangenome and structural-variation analyses which show the presence/absence variants and homeolog-specific expression in allotetraploid cotton, which justifies the existence of population-specific or environmentally unstable QTLs [36]. Lastly, the historical hypothesis of an unavoidable oil–fiber trade-off does not hold any longer: time-course omics and tissue-specific engineering demonstrate that antagonism is situation-dependent and may be alleviated through time-based or tissue-specific control and enhancing the overall strength of the source plant [37,38]. Collectively, contemporary datasets validate the pathway core and shift breeding and engineering approaches to systems-level flux management, regulatory timing, and pangenome-conscious marker design. The developmental and environmental responsive seed-specific promoters and cis-regulatory elements provide a means by which oil characteristics may be altered very specifically. Also, gene expression timing is of paramount importance in cotton because the coinciding processes of fiber maturation and seed growth pose unique constraints when it comes to source and sink optimization and trait uncoupling as a part of any breeding programme [3,39]. The timing of gene expression is also vital in cotton since major developmental events coincide: fiber elongation (0–20 DPA) and secondary wall thickening (16–40 DPA) are simultaneously accompanied by the commencement of embryo reserve accumulation and triacylglycerol (TAG) biosynthesis (15–40 DPA) [40,41,42]. These pathways share the same assimilate and precursor pools (sugars, acetyl-CoA, ATP), forming physiologically connected source–sink limitations. In addition, a number of regulation pathways, such as ABA signaling and seed-development transcription factors LEC1, WRI1, ABI3, and FUS3, modulate fiber and seed metabolism [43,44]. Effective trait uncoupling therefore requires coordinated management of both shared metabolic resources and regulatory networks.

## 3. Genetic Architecture of Oil and Agronomic Traits

**Figure 2:**
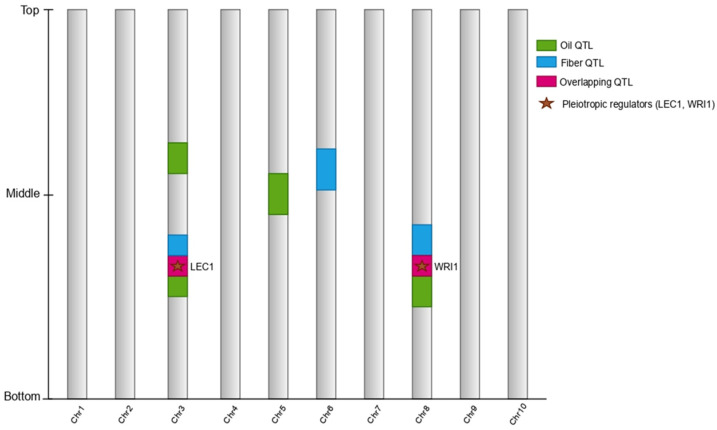
Genetic architecture of cottonseed oil and fiber traits: overlapping QTLs and pleiotropic regulators.

Cottonseed oil content in upland cotton (*Gossypium hirsutum*) is a typical quantitative trait controlled by many genes, each contributing small to moderate effects, and influenced by both lipid-pathway genes and the source–sink dynamics of the plant. Recent linkage mapping, genome-wide association studies, and systems analyses describe a scenario in which oil-related genes are frequently proximal to or overlapping with genes controlling seed size, lint yield, and fiber quality, making correlated responses to selection more common than independent outcomes [45,46] (Figure 2). Many QTLs of seed oil content (SOC) have been identified across bi-parental populations and diversity panels, some of which are consistent across environments and others of which have strong G ×E interactions. For example, a 140-line RIL population grown in multi-environments identified novel SOC QTL and narrowed down relevant intervals, with recessive inheritance of some and diverse oil-soluble fractions [45].

We present a schematic representation of the genomic regions underlying seed oil content (SOC) and fiber-related traits in upland cotton, illustrating overlapping quantitative trait loci (QTLs), pleiotropic regulators, and trait-specific loci across A- and D-subgenomes. Colored bars indicate the direction and magnitude of QTL effects on oil and fiber traits, while arrows denote pleiotropic or tightly linked genomic regions influencing both traits. Candidate regulators involved in carbon allocation, lipid biosynthesis, and seed development (e.g., *WRI1* and *LEC1*) are highlighted to illustrate shared genetic control points. The clustering of lipid- and energy-related hub genes and their association with SOC is supported by linkage and association analyses reported by [47], demonstrating that energy-flow and lipid-biosynthesis pathways are organized into functional genomic modules relevant to breeding strategies.

Genome-Wide Association Studies (GWAS) of Cotton SNP63K/80K arrays show localized SOC with dozens of SNPs widely distributed throughout the A and D sub genomes; many coincide with lipid-pathway candidates (e.g., acyltransferases, desaturases), supporting a common genetic background between oil accumulation and seed development [48,49]. In parallel, recent work in QTL mapping of seed traits by [50] identified over 130 seed-related QTL in a *G. hirsutum × G. darwinii* CSSL population, including regions that overlap with oil-related loci and explain 6–18% of phenotypic variance in some environments. Linkage studies have also reported major-effect loci (e.g., qOil-3), which indicates that despite the polygenicity of SOC, larger-effect QTL can be identified to assist in marker-assisted introgression [51]. For example, some of these major-effect QTL in modern studies show LOD values > 7–10 and phenotypic variance explained (PVE) over 10%, suggesting real practical utility. The genetic architecture is further supported by GWAS summary data reporting R^2^ values of ~4–9% for individual SOC-associated SNPs [8]. Meta- and integrative mapping allows us to identify two useful categories of regions: (i) nearly identical regions that influence both SOC and yield/oil traits (facilitation or antagonism), and (ii) H/R regions that reflect both clean opportunities to enhance oil without lint cost and clean opportunities to increase lint without oil cost. Network-based approaches, such as Weighted Gene Co-expression Network Analysis (WGCNA), have begun to prioritize candidates within these regions, for example, a glycosyl-hydrolase (*GhHSD1*) related to oil-accumulation networks, demonstrating how network context can be used to distinguish between shared and trait-specific nodes [46] (Table 1).

The primary limitation of pleiotropy is that a single gene can code for only one trait. Regulators of seed development/seed maturation (the LAFL module: *LEC1*/*LEC2*/*FUS3*, plus *ABI3*) and the master regulator of lipid metabolism, *WRI1*, together coordinate not only glucose as a carbon distribution resource but also seed morphology and composition. Their activity can be used to explain the correlated change in oil, protein, and seed size noticed in the mapping populations [57,58]. There is now converging evidence for cotton-specific functional roles: seed-specific expression of a cotton *AtWRI1* transgene, in *G. hirsutum*, increased seed oil content, reinforcing *WRI1* as a point of leverage in pleiotropic networks [19]. Furthermore, historical prioritization of lint yield during domestication and modern breeding may have reinforced unfavorable correlations by reducing allelic diversity at oil-related loci. This selection pressure has likely increased the incidence of linkage drag in elite cultivars, thereby constraining simultaneous improvement of oil traits [8,59].

## 4. Trait Interactions: Phenotypic and Genetic Correlations

The relationships among traits that determine cottonseed oil content (SOC) arise from both genetic factors and whole-plant source–sink physiology during boll development and seed filling. Correlations among SOC, lint yield, seed weight, and seed protein in cross-mapping panels and breeding populations are influenced by background and environment and vary in sign and magnitude, indicating polygenic control and G × E interactions. However, there are several stable patterns.

### 4.1. Oil vs. Lint Yield

**Figure 3:**
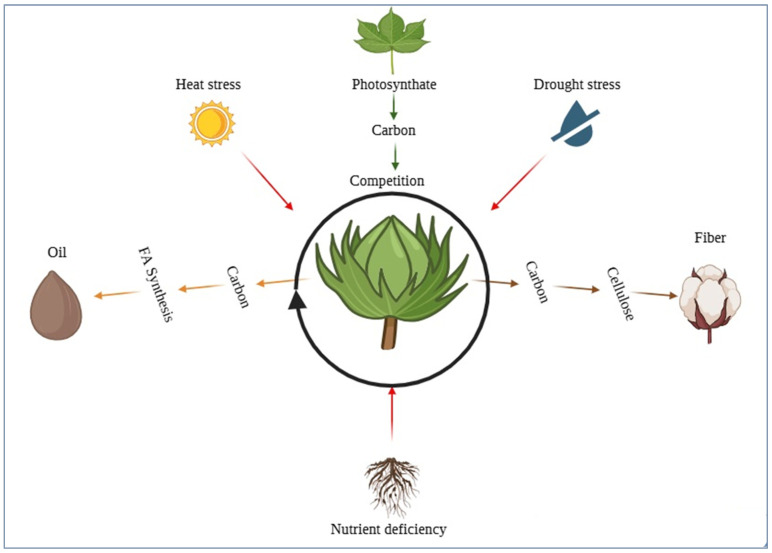
Source–sink competition between seed oil accumulation and fiber cellulose deposition during cotton boll development.

It has long been expected that there is competition between developing fibers and seeds for assimilates. Physiologically, as the boll load increases, assimilate supply is tightened and there occurs a change in the relative balance between the source strength (photosynthesis) of the plant and the competing sinks (fibers, embryos) [37,60] (Figure 3). Empirically, reports on the relationship between SOC and lint yield remain inconsistent across studies, with some datasets showing negative correlations and others reporting neutral or even positive associations [61] (Table 2). In a multiple-environment selection experiment, both lint and seed yields have had relatively little or unimportant correlations with oil and certainly not the strict genetic antagonisms that may be expected; hence, at least in carefully selected environments and germplasm, increases in both yields are quite possible [62]. However, this contrasts sharply with other germplasm surveys and path-analyses that have documented significant negative associations between seed cotton yield and SOC, supporting the classic hypothesis of competition for assimilates [37]. These conflicting findings indicate that the relationship is neither universal nor purely genetic but emerges from complex interactions among genotype, management, and canopy photosynthesis dynamics.

Photosynthates produced in the leaves are transported into developing bolls, where they serve as a shared carbon pool for two major sinks: (i) fiber development, which requires carbon for cellulose biosynthesis, and (ii) seed oil accumulation, which uses carbon precursors for fatty acid (FA) synthesis. Abiotic stresses such as heat and drought, as well as nutrient deficiencies, reduce photosynthate supply and intensify competition between these sinks. Concurrently, other germplasm surveys and path-analyses have found negative associations between seed cotton yield and SOC, which is in line with competitive interactions in certain genetic backgrounds or management systems. The divergence among studies also reflects differences in boll retention patterns, environmental stress levels, and the timing of seed vs. fiber growth, meaning that correlations observed in one breeding population or environment may not extrapolate to broader production systems. Thus, whether oil and lint are antagonistic traits remains an unresolved controversy in cotton breeding, driven in part by inconsistencies in QTL stability, trait plasticity, and G × E responses. The differences highlight the role of environment, boll load, and canopy photosynthesis in defining realized correlations [63,64]. Recent carbon-flux partitioning models and ^13^C-isotope tracing experiments provide direct physiological support for these observations: under high boll load or abiotic stress (heat, drought), isotope tracing and metabolic assays show reduced ^13^C incorporation into fatty-acid pools and a relative increase in carbon allocated to structural/elongation processes (fiber), consistent with a reallocation of assimilates away from oil biosynthesis under source-limited conditions [65,66,67]. Breeding schemes can prevent a universal reduction in lint by targeting SOC loci that are unrelated to Yield-TQTLs and by controlling source–sink during selection nurseries (e.g., stress mitigation, optimal fruit-fruiting) [37]. However, there is little evidence of identifying SOC QTLs that stay constant across environments, and even stable QTL for oil content have been reported in other experiments to change the effect size when subjected to stress conditions [68], further adding to uncertainty about the extent to which oil-producing and oil-utilizing cotton ideotypes may represent physiological tradeoffs.

**Table 2:** Phenotypic correlations between SOC and other seed/crop traits.

Trait Pair	Correlation (Type)	Strength (r-Value or Qualitative)	Reference
Oil vs. Lint yield (total lint kg/ha)	Generally neutral to weak/context-dependent; both weak positive and weak negative associations reported	r ≈ 0.00–0.08 (weak/near-zero in multi-germplasm surveys); some biparental studies report negative genetic correlations in specific crosses.	[69]
Oil vs. Lint % (lint percentage of seed cotton)	Typically weak/variable; can be weakly positive in some materials and weakly negative/neutral in others	Qualitative: weak/inconsistent across panels (no consistent large)	[70]
Oil vs. Seed protein (%)	Usually negative or weakly negative, but exceptions exist (depends on germplasm)	Reported r values vary: weak negative to weak positive depending on panel (e.g., RBTN r = +0.34; Pee Dee r = −0.02; calibration r = −0.25)—overall trend = weak negative in many studies.	[71,72]
Oil vs. Seed index/Seed weight (seed size)	Positive (larger seeds often contain more oil by mass)	Examples: r = 0.88 reported in some genotype panels (strong positive); other surveys report positive but lower correlations (moderate)—qualitative summary: positive, strength variable.	[73]
Oil vs. Seed-cotton yield (kg/ha)	Mixed: can be positive (when seed size increases without lint penalty) or negative (if higher lint diverts assimilates)—often context-dependent	Qualitative: variable (studies report both weak positive and weak negative associations depending on genotype/environment)	[62]
Oil vs. Fiber quality traits (strength, length, micronaire)	Mixed—some studies show weak positive associations with certain quality metrics (length, strength) in some germplasm; others report no relation or antagonism	Qualitative: weak/trait- and germplasm-dependent (e.g., some reports of positive relationships for fiber strength or length; other surveys show no consistent pattern)	[72]
Oil vs. Fatty-acid profile (e.g., oleic:linoleic ratio)	Not a phenotypic correlation per se—composition and total oil are related but controlled by different loci; composition can be altered independently by major genes (e.g., FAD2)	Quantitative: editing FAD2 shifts composition dramatically (e.g., oleic ↑ from ~18% → ~75–77%) while total oil % may remain largely unchanged. So composition change ≠ consistent change in total SOC.	[18]
Oil vs. Abiotic stress response (heat/drought effects on SOC)	Environmentally mediated; generally negative under stress (stress often reduces source strength and can alter fatty-acid proportions)	Qualitative: G × E significant—high temperature during seed filling can shift FA composition (more saturated), and drought can reduce total SOC or change composition; strength depends on environment & genotype.	[62]

### 4.2. Oil vs. Seed Weight and Protein

The dominance of embryo reserves means that SOC is frequently correlated with seed size and seed index but frequently negatively correlated with seed protein, which reflects biochemical storage pathways of carbon vs. nitrogen. A synthesis chapter on cottonseed oil genetics reports negative oil-protein correlations and associations with yield components (seed cotton yield, lint %, seeds/boll), illustrating how storage networks compensate for reproductive allocation [74]. Large-scale GWAS (*n* = 500 accessions; eight environments) yielded extremely high broad-sense heritability of SOC (H^2^ = 80.97), with stable genotype rankings derived from marker-based prediction [75]. These findings indicate that, although oil–protein correlations exist, SOC can be directly and effectively selected using genomic tools.

Comparative omics during seed filling indicates that carbon partitioning pathways (glycolysis, fatty acid/TAG assembly) co-vary with oil accumulation, whereas shifts towards phenylpropanoid/secondary metabolism can antagonize oil deposition—an indication of the mechanism behind observed trade-offs with other seed constituents [21,53]. This molecular pattern is reinforced by isotope-pulse studies showing that genotypes and conditions with elevated flux into phenylpropanoid or secondary-metabolism pathways exhibit correspondingly lower ^13^C enrichment of acetyl-CoA and fatty-acid pools, providing direct evidence that diversion of carbon skeletons can limit TAG biosynthesis in seeds [67,76]. Whether direct index selection on SOC and seed size can be effectively conducted is an open question because, as noted, antagonism between oil and protein can be the limiting factor, and perhaps more editing or selective editing/regulation of master nodes to favor TAG production without also reducing amino acid/N assimilation is necessary [53].

### 4.3. Fiber Traits vs. Seed Traits

Fiber initiation and elongation take place on the seed epidermis, which precedes embryo reserve accumulation. Reviews of cotton source–sink physiology highlight that when boll load is high, assimilate competition escalates; any environmental stress (heat, drought) decreases source strength, augmenting trade-offs between fiber growth and seed filling [37,60] (Figure 3). Dynamic canopy-assimilation modeling and within-canopy partitioning studies show how spatial and temporal patterns of light interception and photosynthetic capacity translate into variable carbon supply to bolls; coupled isotope partitioning experiments confirm that drought or heat shifts the balance of assimilate allocation to-ward maintenance and cell-elongation sinks (fiber), at the expense of oil biosynthesis in developing embryos [65,77,78]. On the genetic side, fiber-quality GWAS on multi-environment trials reveal plentiful loci with small effect and extensive G × E, which recapitulate SOC architecture. Co-localization of QTL fiber traits, seed size, and SOC contributes to understanding how favorable responses to these traits may be correlated but antagonistic in other haplotypes [79]. Additional network-directed analysis indicates that there can be modules associated with lipid metabolism that are independent of (or partly overlap with) fiber-development modules; finding non-overlapping loci that are evolving under selection due to fiber-enhancement efforts thus has the potential to decrease genetic drag on regions that are under selection by fiber-improvement strategies.

A combination of physiological and genomic intervention would seem to be suitable to mitigating fiber to seed trade-offs. The goals of physiological management should be to maintain source strength through enhancement in photosynthetic capacity, adequate efficiency of nutrient and water use, and reduction in effects of environmental stress. At the same time, genomic studies should aim at better prediction of SOC-related loci and regulatory modules to separate fiber elongation and seed reserve deposition. This kind of physiology-genomics integration would improve the assimilate availability, but the genetic factors could be fine-tuned selectively, and the resultant balance between fiber quality improvement, seed development, and yield stability could be achieved.

### 4.4. Physiological Trade-Offs Between High-Oil Genotypes and Abiotic Stress Responses

Although the primary focus of this review is the genetic and molecular basis of cottonseed oil improvement, it is important to briefly consider whether elevated oil accumulation interacts with abiotic-stress adaptation. To date, no studies have directly evaluated whole-plant stress physiology in high-oil–specific cotton genotypes; however, several lines of evidence suggest that oil-related pathways may intersect with drought- and salinity-response networks.

Seed oil biosynthesis is closely associated with carbon distribution and energy condition, which are both vulnerable to water-deficit and heat stress. The events of drought at the boll filling stage lower the supply of assimilates and may repress fatty-acid biosynthesis genes, including *WRI1*, *FAD2*, and acyltransferases [80]. Multi-environment transcriptomic comparisons have shown that naturally higher SOC genotypes have increased down-regulation of lipid biosynthetic pathways in response to drought relative to moderate-oil genotypes [81], potentially placing them at a disadvantage during stresses where sink demand surpasses source capacity. Physiological studies in cotton have established that high-oil accessions sometimes show modified stomatal behavior, altered osmolyte accumulation, or increased antioxidant activity under drought [82,83], although these studies evaluated genotypes with variable oil content rather than explicitly bred high-oil lines. Such traits may reflect compensatory adjustments in carbon partitioning and photo assimilate transport, but direct causal links between elevated SOC and stress physiology remain unresolved.

Lastly, salinity stress has been reported to influence the seed oil composition in various oilseed species and has been known to cause changes in carbon metabolism and lipid remodeling. Similar trends of stress-induced unsaturation and TAG assembly modification were seen in cotton [84], which is why high-oleic or high-SOC genotypes may react to ionic imbalance in different ways, although experimental evidence is not available. In general, there is some evidence of the possibility of interactions between high-oil phenotypes and abiotic-stress responses, but the field has not been systematically tested in terms of controlled comparisons of high- vs. moderate-oil genotypes. This is a crucial area of knowledge gap and future research focus that is critical in coming up with climate resilient and dual-purpose cotton ideo-types.

## 5. Molecular Insights from Omics

**Figure 4:**
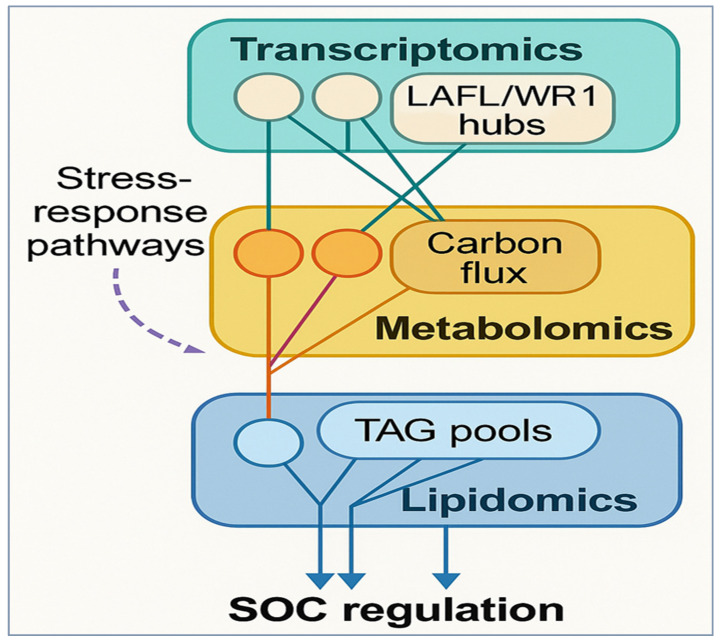
Multi-omics integration for cottonseed oil improvement. Colors denote omics layers (transcriptomics: green, metabolomics: yellow, lipidomics: blue); solid arrows indicate regulatory or metabolic flow, and dashed arrows indicate indirect or stress-mediated effects contributing to SOC regulation.

The breakthrough in high-throughput omics, including transcriptomics, co-expression networks, metabolomics, and lipidomics, among others, has significantly enhanced our understanding of the molecular network that regulates cottonseed oil content (SOC) and interacts with major agronomic traits. The similarities and differences in the approaches demonstrate that regulatory networks, biochemical pathways, and carbon flux dynamics all interact to define SOC and the tradeoffs associated with fiber and seed traits. Transcriptomic profiling has emphasized the key role of seed maturation regulators in the process of oil biosynthesis. During mid-to-late embryogenesis, the LAFL transcription factor complex (LEC1, ABI3, and FUS3) is strongly induced, which correlates with the peak period of TAG deposition and integrates both TAG and protein synthesis [52] (Figure 4). *WRI1* (WRINKLED1) is a central transcriptional activator of fatty acid metabolism: seed-specific expression of *AtWRI1* in *Gossypium hirsutum* resulted in an impressive ~35% increase in seed oil content, accompanied by a fourfold increase in oil body number [19]. A genome-wide search in cotton identified 22 *WRI*-like genes, whereas in vivo characterization of one of them, *GhWRI1a*, demonstrated that it can confer increased oil synthesis in heterologous model plants, confirming its conserved gene regulatory role [52].

Despite the central role of transcriptomic datasets in the identification of seed-development regulators, it is becoming clear that timing of sampling, particularly in relation to the Days Post Anthesis (DPA) at which tissues are sampled, may have a powerful influence on inferred expression dynamics. Recent RNA-seq experiments indicate that a 2–3 DPA shift alone can alter the apparent peak of LAFL- WRI1 activity and downstream lipid-pathway gene induction (e.g., variation around 18–24 DPA sampling windows), a fact that may partly explain discrepancies between datasets that suggest some metabolic genes are not always a module. Also, experiments produced by other sequencing systems or library preparation chemistries can be associated with a small bias without cross-platform normalization (e.g., TMM, RUVseq). This has caused an increased focus on combined meta-transcriptomics to provide strength of candidate-gene inferences across platforms and experiments [85,86].

Candidate gene identification has further benefited from co-expression network analyses, especially WGCNA. An integration of QTL analysis and network data identified *GhHSD1*, a glycosyl hydrolase, as a novel regulator of oil biosynthesis. Transgenic *Arabidopsis*, when overexpressed, showed an increased seed oil content, indicating that *GhHSD1* indeed has a functional role in oil biosynthesis [46]. These oil-associated networks frequently overlap with hormone signaling pathways, particularly abscisic acid (ABA) and auxin, highlighting multilayered regulatory coordination between development and metabolism [53,87]. In addition, genome-based studies indicate that cotton has clusters of genes involved in lipid metabolism, such as desaturases and acyl-CoA synthetases, which are also preferentially expressed in seeds, suggesting co-regulation that supports the requirements of fiber elongation and oil storage [46].

Metabolomic and lipidomic methodologies can be used as complementary biochemical validation. Comparative studies of high- and low-oil accessions of cotton reveal that high-oil genotypes possess elevated pools of glycolytic intermediates and fatty acyl-CoA, whereas low-oil genotypes redirect carbon toward secondary metabolic pathways, including phenylpropanoid and flavonoid biosynthesis [53]. Lipidomic profiling reveals strong intraspecific variation in TAG composition, especially in the oleic-to-linoleic ratio related to variation in the FAD2 locus. In upland cotton, knockout of *GhFAD2-1A/D* with CRISPR/Cas9 significantly increased oleic acid content (approximately 75–77%) and decreased linoleic acid without adverse impacts on fiber quality or seed germination [8,18,88]. Studies involving isotope labeling further indicate that conversion of sucrose to hexose is a key metabolic bottleneck that determines whether assimilates are channeled into fiber extension or oil production, providing insights into the physiology underpinning trait trade-offs [21].

Collectively, these integrated omics datasets depict SOC as a systems-level trait controlled by interacting regulatory layers. In one example, a GWAS of more than 500 accessions across multiple environments revealed extremely high heritability of SOC (H^2^ = 0.966), yet significant portions of the variation were driven by transcriptional divergence at metabolic hubs [75]. Further, the modules of lipid biosynthesis are often found to overlap with stress-response mechanisms, explaining why SOC incur abiotic stresses [53] (Figure 4). In short, omics technologies come together to form a story in which master regulators such as WRI1 and LAFL factors, integrate seed development and oil biosynthesis. Co-expression networks indicate the interplay with hormonal and developmental signaling, while resource allocation and variability in composition are elucidated by metabolomics/lipidomics. SOC thus emerges as an emergent property of coordinated regulatory architecture, supporting the application of systems biology frameworks to simultaneously improve oil content and fiber performance in cotton.

## 6. Breeding Challenges and Trade-Offs

Advances in fiber yield and quality have routinely outpaced advances in cottonseed oil content and quality due to a combination of biological, technical, and institutional obstacles. The renewed optimism offered by advances in molecular tools and underlying genomic resources notwithstanding, seed oil traits and their interaction with fiber-related traits are complex and put genetic enhancement under particular strain.

### 6.1. Genetic Bottlenecks and Limited Diversity

Among the most urgent limitations is the existence of a small genetic base of upland cotton (*Gossypium hirsutum*). Historically, modern breeding programs have focused on fiber yield, lint percentage, and staple length at the expense of seed-related traits. This long-standing selection bias has resulted in reduced allelic diversity at oil-associated loci, thereby constraining breeders’ ability to exploit natural variation [8]. Although wild and semi-domesticated cotton species such as *G. barbadense*, *G. arboreum*, and *G. herbaceum* exhibit higher seed oil content and more favorable fatty acid compositions, the introgression of these traits is hindered by reproductive incompatibilities and linkage drag. In *G. barbadense*, transfer of high-oil alleles is frequently associated with the co-introduction of undesirable fiber or other agronomic traits that preclude their use in commercial breeding [74].

### 6.2. Antagonistic Trait Correlations

The greatest challenge has been the negative relationships between seed traits of interest and important agronomic or seed quality traits. An example of this is the inverse relationship between oil and protein concentration in cottonseed, as they compete for access to the carbon and nitrogen supply during the seed-filling period [49]. Similarly, an increase in lint percentage, one of the principal determinants of fiber profitability, is associated with reduced seed index (seed size/weight), which subsequently compromises oil yield/hectare [89]. These tradeoffs are not absolute but are due to more complex pleiotropic interactions as well as to linkage relationships. In recent studies of QTL in multi-parent advanced generation inter-cross (MAGIC) populations, it has been confirmed that there exist some alleles that both positively and negatively affect lint yield and oil content simultaneously [90]. Therefore, it is one of the breeding challenges to decouple these correlations.

### 6.3. Phenotyping Limitations

Cottonseed oil content cannot be assessed visually and must be quantified through destructive sampling and chemical analysis. Conventional systems, e.g., Soxhlet and gas chromatography, are precise but time-consuming, labor-intensive, and cumbersome in massive screening efforts in the primary half-breed generation. Near-infrared spectroscopy (NIRS) has been proposed as a fast and non-destructive alternative, but its use remains limited to date, with calibration across genotypes and environments being required [70]. This bottleneck of phenotyping has led to a situation where oil properties are rarely included in the majority of breeding pipelines, which have only served to marginalize the property further. Consequently, breeders seldom record the seed oil dynamics on the same selection trials that they are noting fiber traits.

### 6.4. Genotype × Environment Interactions

Genotype × environment (G × E) interactions are observed with substantial levels of cottonseed oil content due to the fact that the mechanisms underlying fatty-acid biosynthesis are strongly coupled to the overall carbon balance of the plant, which varies significantly between environments. Figure 3 shows that the development of bolls is dependent on a common pool of assimilates that need to be divided between competing sinks (i.e., fiber cellulose synthesis and seed oil accumulation). Oil content is therefore altered by any environmental factor that changes carbon supply or sink strength despite having strong intrinsic potential in the genotype.

The effect of environmental stresses like heat, drought, and nutrient deficiency disrupts photosynthesis, phloem loading, and assimilate transport and makes less carbon available to both fiber and lipid pathways. Increased temperatures during boll filling, as in the case of fatty-acid desaturation, favor saturated fatty acid accumulation at the expense of nutritional value of the oil [60]. Drought stress also limits the carbon reaching the seeds, commonly decreasing triacylglycerol (TAG) accumulation unpredictably against fiber development since the formation of cellulose can be held intact more in carbon-restricted environments [91].

On the other hand, favorable environments having sufficient supply of water and soil fertility can lead to higher supply of total photosynthates, but relative partitioning of fibers and seeds can differ across genotypes based on sink strength, flowering, or boll load. This is the reason why the genotypes that perform remarkably well in controlled or irrigated conditions often demonstrate inconsistent oil content in the situations managed by farmers or those susceptible to stress.

Since the mean performance as well as the stability of SOC is determined by these environment-sensitive physiological processes, breeding progress has become a difficult issue. To enhance stability, it is not only necessary to choose the best alleles in lipid biosynthesis but make sure that such genotypes have a stable source designed to respond to the changing field conditions and a balanced sink demand [92].

### 6.5. Institutional and Breeding Priorities

Although the use of cotton as a dual purpose crop has been recognized as having great potential, institutional breeding goals have been predominantly fiber-based, with seed oil and seed nutritional characteristics being considered as secondary or tertiary objectives. Traditionally, the breeding program, whether public or private, has been judged and financed mostly on the basis of lint yield, fiber quality, and yield stability [93,94]. Consequently, there has been a low level of investment in phenotyping and selection of seed-quality characters.

This structural focus on fiber has been supported by market economics and institutional testing structures. Fiber is the most important source of revenue in most cotton value chains and most national variety release systems explicitly focus on lint yield, fiber strength, micronaire, staple length, and ginning outturn. The seed oil content, seed protein, and fatty-acid composition are rarely listed as compulsory test parameters [95,96]. As a result, oil characteristics are rarely quantified on a regular basis, postponing the application of the available information on genetics into practical breeding pipelines.

Simultaneously, genomic and molecular resources pertaining to lipid biosynthesis have become accessible in substantial amounts: QTL and meta-QTL maps of SOC, marker-trait links of GWAS and transcriptome-scale characterization of TAG biosynthetic pathways [11,48]. The findings however, are not used fully due to the fact that operational breeding schemes seldom incorporate SOC into the selection indices at early generations. The majority of breeding programs prioritize lint performance up to F6, and seed-quality traits are either not assessed at all or only assessed in late-generation or pre-release trials, which have often lost much genetic variation by this point [8,95]. Country-level examples reinforce this institutional bias: in China, targeted efforts to improve cottonseed quality have been introduced, but fiber yield still dominates formal breeding objectives [97]; in India and Pakistan, ginning outturn, fiber length, and staple strength remain dominant criteria in national performance trials, with SOC absent from routine evaluation protocols [98]; and in the United States, oil composition and content are measured mainly in specialized research programs rather than in mainstream breeding pipelines [55].

In general, these structural, economic, and institutional reasons can explain the lack of proportional gains on breeding with cottonseed oil, even though it has strong genetic foundation, and genomic tools exist to help in breeding. The dual-purpose potential of cotton as a fiber- and oil-producing crop will not be fully utilized without a specific financial support, routine SOC phenotyping, and the inclusion of oil properties into the variety release criteria and selection indices.

To facilitate reducing these institutional and technical constraints, coordinated international breeding consortia might help dramatically increase the incorporation of seed oil characteristics into mainstream cotton improvement. High-throughput phenotyping centers with standard NIRS and lipidomic calibration pipelines would minimize cross-laboratory and allow for vast-scale and comparable measurements of oil properties across breeding schemes [99]. Similarly, large data repositories, combining genomic, phenotypic, and multi-environment trial data, as in other crops recently carried out, would allow for strong modeling of G × E interactions and allow breeders to collectively assess germplasm across regions [100,101]. These types of collaborative models would enhance access to a wider range of germplasm, decrease redundancy in resource-constrained programs, and eventually enable the incorporation of oil traits to be better organized into national and international breeding goals [102].

## 7. Strategies to Bridge the Divide

The delivery of cotton ideotypes that combine high-quality fiber with improved seed oil requires an integrated set of strategies that aim to (i) address antagonistic correlations, (ii) expand the exploitable genetic base, and (iii) speed up selection in real-world settings. We summarize the progress in multi-trait genomics, pre-breeding using exotic/wild germplasm, precise genome editing, and systems-level design below, as well as practical observations on how these can be applied in breeding programs.

### 7.1. Multi-Trait QTL Dissection and Genomic Prediction

Multi-trait QTL mapping helps distinguish between pleiotropy and tight linkage, and determines when oil and fiber loci can be separated by recombination and when regulatory modification is necessary. Multi-environment GWAS of SOC has identified QTLs across multiple chromosomes (e.g., A- and D-subgenomes), which offer selection marks and a framework to fine-map and then validate a candidate gene or locus [46]. Recent quantitative-genomics studies further quantify the extent to which selection on lint percent, seed index, lint yield, and SOC often exhibits inverse but generally weak-to-moderate correlations, which can be overcome through index-based or multi-trait models [56].

Genomic selection (GS) is particularly appealing for SOC improvement, since it minimizes reliance on destructive phenotyping and facilitates multi-trait prediction under G × E interaction. Public breeding program reviews describe operational pipelines (training population design, cross-validation under target environments, retraining cadence), and conclude that GS could expedite cotton gains and still incorporate pre-breeding diversity [55]. With decreasing costs of genotyping and training resources that span across environment, breeders can apply multi-trait, multi-environment (MT-ME) models to optimize trade-offs between SOC, fiber quality, and yield stability.

### 7.2. Widening the Genetic Base: Pangenomes and Pre-Breeding Resources

The recent assemblies of the pangenome and multiple references demonstrate that the upland cotton gene pool includes significant structural variation and presence/absence variants that are not seen by single references—variation that overlaps with domestication sweeps, introgressions, and agronomic loci [103,104]. A recent pangenome-scale study highlights the fact that graph-based representations can be more useful for estimating the allelic diversity underlying yield and fiber characteristics, which are also potentially SOC-relevant and deployable resources [105].

The translation of this variety into elite backgrounds is based on pre-breeding populations. Chromosome segment substitution lines (CSSLs) provide near-isogenic windows to identify small-effect QTL between seed and fiber traits to uncouple linked loci; inter- and interspecific CSSLs and novo sets based on *G. tomentosum* indicate results of tractable introgressions on seed-related traits [106,107]. Nested association mapping (NAM) is a complement to CSSLs, combining GWAS-like resolution with family-based power in complex traits, providing a path to the decomposition of SOC-related variation and estimation of pleiotropic load [108,109]. Collectively, pangenomic discovery + CSSL/NAM validation shorten the route between locus discovery and breeder-ready haplotypes, including genotypes in which oil increases without compromising fiber performance.

### 7.3. Precision Genome Editing to Break Unfavorable Linkages

CRISPR/Cas provides specific modifications that increase the production of oil quantity and quality with minimal collateral implications on fiber. Knockout of *GhFAD2-1A/D* in allotetraploid cotton resulted in non-transgenic, high-oleic lines (~75–77% oleic acid) with consistent agronomic performance, and the example of a clean pathway to enhance oxidative stability of cottonseed oil [18]. The oil profiles and protein trade-offs are also regulated by the natural or artificial alleles of FAD2, which provide an allelic series for tailoring fatty-acid composition [110]. Parallel seed-specific up-regulation of *WRI1* (or editing of its cis-regulatory motifs) boosts flux through glycolysis and fatty acid biosynthesis (recent seed-targeted expression of *AtWRI1* in cotton boosts SOC, supporting WRI1 as a lever without apparent fiber penalty) [19]. In the future, pathway flux can be tuned exclusively in seed tissues through regulatory (promoter/enhancer) editing, base/prime editing, and multiplexed edits (e.g., stacking WRI1, DGAT, and acyl-editing enzymes), and without pleiotropic effects on fibers. Theoretical and experimental research in the field of plant metabolic engineering provides these approaches to oil crops and is becoming more malleable to cotton [104].

### 7.4. Integrating Exotic/Wild Alleles

Individual wild and exotic Gossypium accessions possess greater SOC and new fatty-acid profiles but are associated with linkage drag and incompatibilities. Reviews and case studies have demonstrated that a stepwise method, (i) QTL discovery in interspecific populations, (ii) validation using CSSLs/NAM, (iii) haplotype-based selection in backcross schemes, and (iv) targeted CRISPR-mediated elimination of residual drag, can generate breeder-ready haplotypes at a pace that exceeds classical backcrossing approaches [8,106]. Multi-reference genomes of high quality now assist in the identification of structural variants that are transmitted by wild donors, enabling selection against deleterious blocks and retention of desirable SOC alleles [111].

### 7.5. Systems Biology and Source–Sink Optimization

Since SOC competes with lint for assimilates, system models that couple transcriptional control, metabolite pools, and carbon flux are necessary to design edits and selection indices that do not compromise fiber performance. Classical and modern syntheses of source–sink dynamics in cotton define the locations of transport, partitioning, and hormonal control; by combining these with SOC networks (e.g., WRI1/LAFL hubs), direct interventions can be made to reestablish balance in allocation during boll filling [37,112]. Field-based physiology affirms that within-canopy light and water regimes influence carbon partitioning, emphasizing that genetic strategies should be reinforced by targeted agronomic optimization [77]. Multi-omics meta-analyses on cotton germplasm reveal that high-oil phenotypes increase the expression duration and amplitude of oil-gene expression. This lever can be achieved through promoter editing or transactivation in seed tissue [113].

### 7.6. Breeding Pipelines: Phenotyping, Indices, and G × E

The application of G × E implies phenotyping at scale and selection to enable deployment. In modern quantitative models, emphasis is put on constructing multi-trait selection indices where lint, seed, and oil yields are explicitly weighted; recent field analysis quantifies the trade-off magnitudes (e.g., lint percent/ seed index; lint or lint percent/ SOC) and offers templates to optimize indices in line with end-use economics [56]. Multi-environment QTL of ultra-high oil lines on the discovery side demonstrate that stable SOC loci can be revealed through replicated field tests coupled with dense markers [45]. With the implementation of GS, population refresh and environmental covariates are the focus of the guidance at the program level, including cost-effective genotyping, which is essential for SOC because destructive phenotyping is the bottleneck [55].

## 8. Future Directions

The dual-purpose improvement in upland cotton, balancing the productivity of the fiber with that of seed oil, requires future-oriented strategies that transcend the present genomic and breeding innovations. A combination of multi-omics datasets, machine learning methods, climate-resilient breeding pipelines, and innovations in synthetic biology is likely to accelerate the development of cotton ideotypes suited for the 21st century.

### 8.1. Integrating Multi-Omics and Machine Learning

The generation of large datasets obtained through genomics, transcriptomics, proteomics, metabolomics, and lipidomics presents an unprecedented possibility to comprehend the interactions of many traits in cotton. The difficulty is, however, to bring these heterogeneous layers of data into actionable knowledge. Machine learning (ML) and artificial intelligence (AI) are increasingly being applied in plant science to discover concealed patterns, forecast gene–trait correlations, and for precision breeding. ML-based predictive models are under development in cotton to predict oil composition, seed quality, and yield stability, combining SNP variation with expression and metabolite information. For example, deep learning models have already been able to increase the accuracy of genomic prediction of seed traits in other oilseed crop varieties, and such methods are under development to apply to cotton. Recent advances have expanded genomic-selection methodologies beyond traditional Bayesian GBLUP, including Bayesian optimization and kernel-tuned GBLUP, which improve predictive accuracy [114]. Deep learning approaches, particularly CNNs, offer advantages for modeling non-additive genetic effects and can out-perform classical models depending on data structure [115]. Multimodal architectures that integrate genomic markers with high-throughput phenomics, such as imaging traits, further enhance predictive ability in crops [116]. Machine learning models, including deep neural networks and random forests, have already improved prediction of seed-oil and quality traits in Brassica and soybean [116,117] and are increasingly applied to cotton. Integrative cotton studies also show that combining SNP data with transcriptomic and metabolomic profiles enables effective ranking of oil-relevant genes and prediction of genotype performance across environments [118]. Combining multi-omics + ML pipelines in the near future will allow breeders to prioritize candidate genes, simulate regulatory networks, and optimize multi-trait selection strategies for simultaneous improvement in fiber and oil content.

### 8.2. Climate-Smart Dual-Trait Breeding Pipelines

Climate change is a significant threat to cotton production, and increasing temperatures, drought, and salinity stress affect fiber production and oil accumulation. Therefore, climate-smart breeding should become a fundamental part of cotton improvement. The next-generation pipelines will be built to incorporate phenomics platforms, crop models, and genomic selection under multi-environment tests to select the genotypes with balanced fiber and oil productivity when under stress. It has already been established that heat and water stress influence carbon partitioning of lint and seed oil; so, ideotypes of adaptation to climate will require regulation of sources and sinks optimally. Studies have shown that heat and water stress shift carbohydrate partitioning, affecting both lint yield and TAG biosynthesis during seed filling [83,119]. Moreover, high-throughput phenotyping can be used in conjunction with speed breeding and doubled haploids to accelerate generation turnover, allowing dual-purpose cultivars to be delivered faster to meet the changing climate.

### 8.3. Synthetic Biology for Oil Trait Enhancement

Synthetic biology is a revolutionary edge to cottonseed oil enhancement. Synthetic biology offers transformative opportunities for cottonseed oil improvement. In model species and oilseed crops, synthetic promoters, CRISPR-based transcriptional activators, and gene circuit engineering have successfully increased TAG content without detrimental impacts on plant growth [120,121]. The same strategies could be applied to cotton to selectively regulate the deposition of oil in seeds by using regulators such as WRI1, DGAT, and LEC1. Moreover, developments in plastid engineering could permit more effective redirection of the carbon flux to oil accumulation, potentially decoupling oil enhancement and fiber yield limitations. Synthetic biology could also enable the design of cotton as a biofactory to produce specialty oils of nutraceutical fatty acids, extending the economic usefulness of the crop beyond the textile and standard vegetable oil markets.

## 9. Conclusions

Cotton (*Gossypium hirsutum* L.), which has long been considered the most significant fiber crop in the world, is now being actively considered as a dual-purpose species with huge potential to play a role in global edible oil and protein production. However, improvement in cottonseed oil content and quality has been constrained by the historical prioritization of fiber traits, together with inherent genetic, physiological, and breeding trade-offs. This review identifies the multifaceted genetic basis of cottonseed oil characters, regulated by polygenic interactions and compounded by pleiotropy and linkage drag with loci related to fiber. The existence of trade-offs (oil vs. lint yield or oil vs. protein content) is validated by phenotypic and genetic correlation and is to a large extent due to underlying source–sink interaction. Transcriptomics, metabolomics, and lipidomics have identified several central regulatory nodes, such as WRI1, ABI3, and LEC1, that coordinate seed metabolism and carbon partitioning and are potential molecular therapeutic targets for genetic engineering [122]. Despite these advances, major challenges remain, including limited genetic diversity in elite cultivars, antagonistic selection pressures on fiber and seed traits, inadequate high-throughput phenotyping capacity, and increasing climate-related constraints on assimilate partitioning.

The proposed dual-purpose improvement framework can be applied in the breeding programs through four steps that are coordinated operationally. First, the integration of multi-omics data (genomic, transcriptomic, lipidomic) can be performed to rank the candidate genes and regulatory hubs that regulate both the olefinous and fibrous properties of the seed. Second, multi-trait genomic selection (MT-GS) models can be deployed to predict fiber yield, oil content, and key source–sink attributes concurrently, enabling breeders to identify genotypes with favorable combined merit. Third, the high confidence targets that arise as a result of the MT-GS and network analyses can be experimentally validated with CRISPR/Cas9 and CRISPRa/i systems and, thus, prove causality and produce better allelic variants. Lastly, canopy photosynthesis, boll filling, fiber development, and seed characteristics can be quantified in high-throughput phenomics platforms in realistic multi-environment conditions. Collectively, these components form a practical and scalable pathway for translating biological insights into dual-purpose breeding outcomes.

Going forward, integrative approaches including multi-trait QTL mapping and genomic selection, the introduction of desirable alleles into the germplasm of the wild Gossypium, the use of CRISPR/Cas9 and synthetic biology to uncouple negative interactions and systems biology modeling enabled by machine learning can promise to overcome these limitations. In practical breeding terms, these advances will also require economically optimized multi-trait selection indices that explicitly balance improvements in fiber quality and yield with gains in seed oil content. The realization of this potential will entail policy support that makes cotton a dual-purpose crop, as well as investment in advanced infrastructures for omics and phenotyping, an international germplasm exchange, and capacity building in cotton-growing regions. Finally, the development of climate-resilient cotton ideotypes that deliver high-quality fiber and enhanced oil and protein content will reinforce cotton’s status as a truly multifunctional crop, contributing not only to economic growth but also to food security and agricultural sustainability worldwide.

## Figures and Tables

**Table 1:** Genetic architecture of cottonseed oil content (SOC): QTLs, loci, candidate genes.

Chromosome/Linkage Group	QTL/Marker	Associated Trait (Oil %, Fatty-Acid Profile)	Population Type	Reference	Validation Status
A/D subgenomes (multiple loci)	Multiple SNPs/QTL clusters identified across A and D subgenomes (GWAS hits; 28 QTL regions reported)	Seed oil % and related seed composition traits (various loci linked to oil% and fatty acid composition)	Diversity panel/GWAS (multi-environment; CottonSNP arrays)	[49]	Statistical association only (GWAS/QTL)
Chr. (reported major QTL region; “qOil-3”)	qOil-3 (major-effect QTL reported in linkage mapping)	Seed oil % (major effect locus contributing to SOC variation)	Biparental/RIL mapping (multi-environment)	[51]	Statistical association only (QTL)
A/D subgenome — *GhFAD2*-*1A*/*GhFAD2*-*1D*	*GhFAD2* (desaturase gene cluster; homeolog pair)	Fatty-acid profile: oleic ↑, linoleic ↓ (high-oleic phenotype); also impacts oil quality	Functional knockout/genome editing (CRISPR in allotetraploid upland cotton)	[18]	Functionally validated (CRISPR, biochemical phenotype confirmed)
(genome locations vary) — *GhWRI1* (*GhWRI1a*, *GhWRI1b*, etc.)	WRI1 family loci (seed-expressed WRI-like genes)	Seed oil % (transgenic/overexpression increases SOC; regulatory hub for glycolysis→fatty acids)	Candidate gene studies; transgenic overexpression and functional characterization	[52]	Functionally validated (transgenic expression → SOC increase)
(various chromosomes) — *GhDGAT*/acyltransferases	DGAT loci (acyltransferase candidates within oil-QTL intervals)	Triacylglycerol assembly; correlated with higher TAG content and oil %	Co-localization/candidate gene within QTL regions; functional inference from expression/transgenics	[53]	Partial validation (expression + functional inference)
Specific mapped interval(s) reported in integrative mapping studies	*GhHSD1* (glycosyl-hydrolase) — prioritized by WGCNA inside SOC QTL	Associated with oil accumulation (network-prioritized candidate; functional test in *Arabidopsis* increased seed oil)	Integrative QTL × co-expression (WGCNA) with transgenic validation (heterologous assay)	[54]	Functionally validated (heterologous transgenic assay)
CSSL/NAM introgressions (various chromosomes)	Small-effect QTL windows identified in CSSLs & NAM (for SOC and seed traits)	Seed oil %, seed index; disentangles linkage drag vs. pleiotropy	CSSL/NAM populations (interspecific introgressions and multi-founder populations)	[55]	Statistical association only (QTL)
Multi-environment GWAS with high H^2^ lines	Stable SOC loci (environment-stable QTLs reported; high broad-sense heritability for SOC in some GWAS panels)	SOC (stable across environments; useful for selection)	Large GWAS panels (*n* ~ 500; multi-environment trials)	[56]	Statistical association only (GWAS)

## Data Availability

No new data were created or analyzed in this study.
